# Sustainable, resilient food systems for healthy diets: the transformation agenda

**DOI:** 10.1017/S1368980019003112

**Published:** 2019-08-13

**Authors:** Mark Andrew Lawrence, Phillip Ian Baker, Claire Elizabeth Pulker, Christina Mary Pollard

**Affiliations:** 1 Institute for Physical Activity and Nutrition (IPAN) Faculty of Health, Deakin University Geelong, VIC 3220, Australia; 2 School of Public Health, Curtin University Perth, WA, Australia

Sustainable, resilient food systems for healthy diets has been identified as the first of the six pillars for action during the UN Decade of Action on Nutrition^(^ ^1^ ^)^. It is now THE defining issue for public health nutrition^(^ ^2^ ^)^. A sustainable food system is one ‘that ensures food security and nutrition for all in such a way that the economic, social and environmental bases to generate food security and nutrition of future generations are not compromised’^(^ ^3^ ^)^ (p. 12). Resilience refers to the capacity of a food system to achieve this same objective ‘in the face of various and even unforeseen disturbances’ including environmental, economic or socio-political shocks^(^ ^4^ ^)^ (p. 19). Sustainable food systems are essential if we are to nourish a projected global population of nearly 10 billion in 2050 within planetary boundaries^(^ ^5^ ^)^. However, today’s food systems are far from sustainable. Not only are dietary risk factors and malnutrition in all its forms the leading contributors to the global burden of disease^(^ ^6^ ^)^, but also food systems are not operating within some planetary boundaries and are contributing to widespread and potentially irreversible environmental breakdown degradation, including potentially irreversible disruption^(^ ^7^ ^)^. Understanding the impact of population dietary intake has extended beyond health and the ability of food systems to provide sufficient quantity, quality and diversity of safe, affordable and nutritious foods, to interlinkages of diets and food systems with climate change, water and land pollution, deforestation and biodiversity loss and other forms of environmental degradation^(^ ^7^ ^)^. The focus on healthy diets from sustainable food systems connects all parts of food supply chains (from food production to consumption) and the social, economic and environmental outputs of those systems^(^ ^8^ ^)^.

Unsustainable food systems producing unhealthy diets is the status quo, a lose–lose dynamic for both human health and the environment. Nevertheless, in principle there is hope. A system disruption might possibly flip new governance arrangements and policy actions for transforming food systems to win–win have been articulated. Certainly there is evidence to support the premise that ‘a healthy diet is a sustainable diet’ and vice versa^(^ ^9^ ^)^. However, achieving these actions presents a major challenge. Food systems are multidimensional socio-ecological systems which involve many actors with diverse interests and worldviews, impacted by policies from sectors including agriculture, food, health, finance, trade and environment^(^ ^10^ ^)^. This means that some of the win–win actions may struggle to achieve full societal and political acceptance, for example the highly contested issue of meat reduction^(^ ^11^ ^)^. Whether a healthy diet is a sustainable diet will also depend on nuances including the circumstances under which different types of foods are produced and consumed^(^ ^12^ ^)^. Therefore, some trade-offs between various competing priorities will need to be made whereby one consideration is prioritised over others. There is a need to embrace system-wide and integrated approaches to interventions (i.e. multiple policy and programming actions that work synergistically on different components and levels of the system)^(^ ^13^ ^)^.

After decades of relative neglect, the need for healthy and sustainable food systems is now receiving greater political attention by governments, international organisations, business groups and civil society organisations. Over the years *Public Health Nutrition* has taken a leadership role in drawing attention to the topic, stimulating academic debate regarding important considerations and highlighting actions that might be taken. It has done this through the publication of numerous peer-reviewed papers, a special issue^(^ ^14^ ^)^, a supplement^(^ ^15^ ^)^ and a targeted editorial^(^ ^16^ ^)^.

The momentum for change is building as academics, civil society and commercial interests across a variety of sectors come together to address these important and difficult issues. One landmark example of this is the ‘Food in the Anthropocene: the EAT-*Lancet* Commission on healthy diets from sustainable food systems’ report (EAT-*Lancet* report)^(^ ^2^ ^)^ launched in Stockholm in June 2016^(^ ^17^ ^)^. Thirty-seven scientists worked together over two-and-a-half years to build the evidence base for this important report. In an increasingly polarised world where social media influences public understanding of issues^(^ ^18^ ^)^, the attempt to bring together experts from various academic fields for evidence assessment is to be commended.

## The EAT-*Lancet* Commission recommendations

**Fig. 1:**
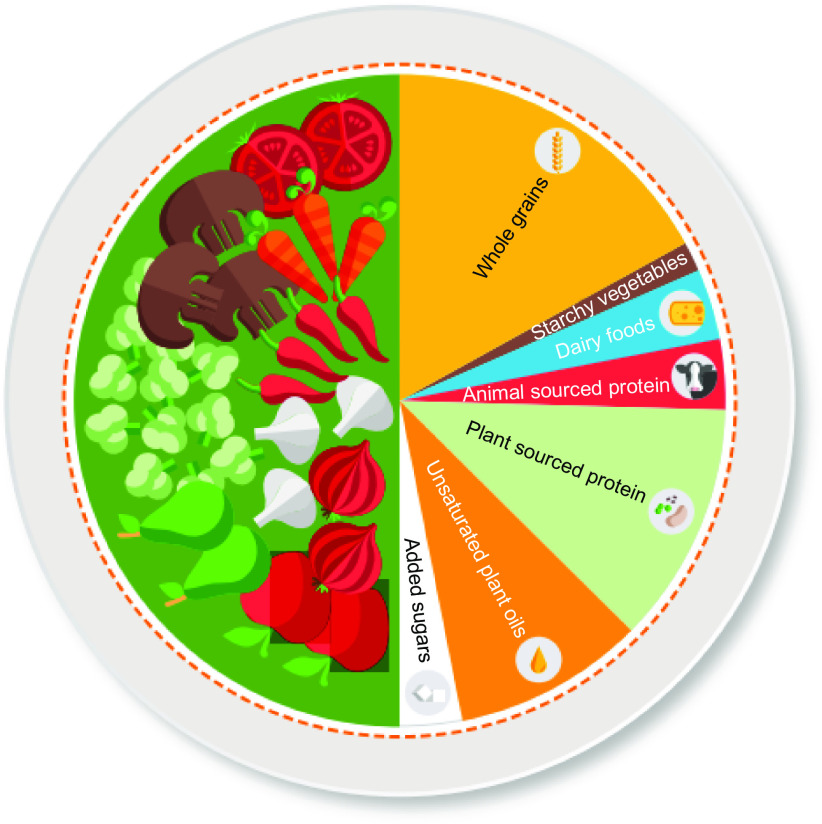
The EAT-*Lancet* Commission planetary health plate

The EAT-*Lancet* report recommendations are predicated on its assessment that there is a current lack of synchronisation between dietary behaviour and its impact on the planet. It recommends that to feed almost 10 billion people in 2050 there is a need for a universal ‘healthy reference diet’^(^ ^2^ ^)^, also referred to as a ‘flexitarian’ diet^(^ ^19^ ^)^. This healthy reference diet contains a diverse range of plant-based foods, low amounts of animal-based foods, unsaturated fats, and small amounts of refined grains, processed foods and added sugars, in amounts appropriate for a healthy weight^(^ ^2^ ^)^. Specific explanatory materials advise that a planetary health plate (for the healthy reference diet; Fig. 1), ‘should consist by volume of approximately half a plate of vegetables and fruits; the other half, displayed by contribution to calories, should consist of primarily whole grains, plant protein sources, unsaturated plant oils, and (optionally) modest amounts of animal sources of protein’^(^ ^19^ ^)^ (p. 9).

The healthy reference diet is designed to be flexible. It accommodates cultural differences, traditional eating patterns and individual dietary preferences. Importantly, the diet is referred to as a ‘starting point’ for the changes needed^(^ ^2^ ^)^. In practical terms, achieving a dietary pattern consistent with the healthy reference diet requires substantial population-level dietary change, particularly for populations following the ‘Western’ dietary pattern. These groups need to consume more than double the amounts of nuts, fruits, vegetables and legumes; half the amounts of red meat and sugar; and significantly reduce food waste.

Five specific multisectoral strategies are recommended to achieve transformation of food systems, to be delivered by ‘an alliance of forces’, including to:

1. seek commitment from a wide range of stakeholders to making significant dietary change;
2. produce better food not just more food;
3. sustainably intensify food production;
4. safeguard land and oceans by stopping land clearing and overfishing; and
5. reduce food losses and waste by at least half.

## The approach

The EAT-*Lancet* report highlights the scale of the challenge. It calls for a ‘multi-level, multi-actor, multi-sector, multi-disciplinary’ (i.e. integrated) approach and an ‘extensive, policy umbrella’ that ‘integrates food, health, and environmental policy into many policy areas, including trade, economics, rural livelihoods, equity, culture, society, and community’^(^ ^2^ ^)^. It emphasises a full range of policy levers from the withdrawal of inappropriate products through to command-and-control regulation, financial incentives and mass communication^(^ ^20^ ^)^. Ideally, governments would unite to lead this type of work as one global strategy.

The report focuses on environmentally sustainable food production and consumption. Surprisingly, other key stages in the food supply chain including food processing and retailing are omitted from the analysis thus far^(^ ^19^ ^)^. Sustainable intensification is recommended as a method for increasing agricultural yields within existing land use and environmental boundaries. However, the report is largely agnostic regarding a preferred approach to food production despite the profound social and environmental implications of the alternatives, for example industrial *v*. agroecological *v*. mixed approaches. Also of note, other sustainability dimensions such as social impact and ethics, including for example equity, food security, labour standards, animal welfare standards and cultural food practices, were similarly outside the scope of work^(^ ^2^ ^)^.

The analysis and recommendations to change the way we eat to feed the world presented in the EAT-*Lancet* report are dramatic but they are not necessarily novel. There have been previous examples of scenario modelling research investigating the implications of dietary practices on health and sustainability albeit usually at national rather than global level^(^ ^21^ ^)^. And the report’s recommendations are broadly consistent with those of an increasing number of national food-based dietary guidelines that are being extended to incorporate sustainability considerations^(^ ^22^ – ^24^ ^)^. What is innovative about the report is the extent of synthesis of available evidence to formulate a recommended dietary pattern and the philanthropic funding that has made resources available to help translate its recommendations into policy and practice. Sophisticated advocacy focusing on key stakeholder engagement and media relations has been used to disseminate the report, amplifying its reach and impact, and generating awareness and support. The scale of global attention achieved to date is underpinned by a series of thirty-eight launch events around the world^(^ ^25^ ^)^, as well as facilitating Food Systems Dialogues which connect diverse food system actors to discuss the issues^(^ ^26^ ^)^.

The Food System Dialogues are one tool to create the impetus for targeted local conversations for food system transformation and help build a shared understanding for agreed action. Other fora where dialogue on healthy and sustainable food systems takes place include the Committee on Food Security, the World Health Assembly, EAT Forum and a number of UN agency platforms. The Food System Dialogues have been held in fourteen countries across six continents with over 900 participants and have generated more than eighty proposals for action. They have engaged stakeholders across sectors, in response to increasing demand and the desire to adopt ‘glocally’ (globally and locally). A mechanism to build capacity of local actors to conduct and report on their own food system dialogues has also been developed.

## The response

Overall, the report appears to have been welcomed by many public health nutritionists and environmentalists as it attempts to bring together the two important issues of dietary health and planetary health. However, it has been opposed by some groups including those with commercial interests, who have questioned the evidence and generated fear that it would cause significant disruption to current food systems (which is one of its core intentions).

There has been a variety of specific criticisms, including: that the recommended diet is a smokescreen for veganism; concerns about the nutritional inadequacy of the recommended diet for population subgroups including pregnant women; and concerns that the flexitarian diet is too prescriptive and unachievable for most people. There was concern about the emphasis on palm oil as an unsustainable crop linked with deforestation, as well as the little attention given to ultra-processed foods. The universal planetary healthy reference diet principles require flexibility in application to ensure they are locally adaptable irrespective of cultural, geographic, social or economic circumstances. That this conceptual work remains to be done at regional, national and local levels means that some critics have struggled to visualise how the recommendations translate into acceptable dietary patterns for specific populations.

## Characteristics of the EAT*-Lancet*report

### Bold in scale

The report is recommending transformation of the food system by the year 2050. The clue is in the sub-heading ‘Great Food Transformation’, which refers to dramatically and swiftly changing all elements of the food system including food production and consumption. This response is critical given the size and scope of the dietary and environmental challenges facing the world and the speed at which environmental instability and disruption is occurring^(^ ^27^ ^)^.

The EAT-*Lancet* report is a more courageous and meaningful endeavour than most policy actions that currently dominate food and nutrition policy debates, and for government will require a return to ‘frank and fearless’ interventions. To date, policy actions targeting diet-related non-communicable diseases and obesity in many countries have focused on consumer-led and market-based approaches. The approaches involve public–private partnerships, information and education directed at individuals, with interventions such as front-of-pack labelling, voluntary reformulation of processed foods, reduction in food portion size and taxes on sugar-sweetened beverages^(^ ^28^ ^)^. Although they can have a role, these actions mostly tweak the minor parameters of today’s dominant unhealthy and unsustainable food systems. They do not address the deeper drivers of today’s dominant food systems model, namely the industrial, exploitative and neoliberal system that incentivises endless market growth and hence ‘consumption to the point of detrimental overconsumption’^(^ ^29^ ^)^ (p. 818). The current market-centric approach may therefore divert attention away from strategies with real potential for change with some even being exploited as a marketing tool for ultra-processed food companies^(^ ^30^ ^)^.

The EAT-*Lancet* report outlines key governance actions for health and Earth systems stewardship and proposes a bold set of integrated policy actions drawing from authoritative and scientific sources. However, achieving a Great Food Transformation presents an immense political challenge only briefly considered in the report. As mentioned earlier, food systems involve many different actors and interest groups often with competing worldviews and beliefs concerning food, health and sustainability, as the diverse responses to the report demonstrate. There are deeply political questions that need to be answered: ‘What issues should be prioritised or ignored?’, ‘What trade-offs between issues are desirable?’ and ‘What roles can and should individuals, industry and government play?’^(^ ^31^ ^)^ Indeed, can achieving the transformative changes being called for realistically be achieved without intense scrutiny of and fundamental changes in the underlying political economies that drive food systems? This is ultimately about understanding and responding to asymmetries in the political, economic and ideological power of different actors within food systems^(^ ^10^ ^)^. This is especially relevant in the context of the massive expansion in the size, global reach and raw market power of transnational food companies^(^ ^32^ ^)^. An important avenue for future investigation would include asking: ‘Who stands to lose and who would gain from a healthy and sustainable food systems future?’ (i.e. for whom is it a win–win or win–lose?) and ‘What does a transformative food systems political economy ultimately look like?’

### Evidence-informed

The EAT-*Lancet* approach aims to build a strong evidence base to inform the dietary targets (mapping the multiple links among food, health and the environment interactions) with sophisticated modelling drawing evidence and advice from across sectors. The approach recognises the need for nuance and cultural appropriateness; for example, it avoids modelling extreme scenarios due to the many contexts and circumstances, including availability of wild animals for food^(^ ^33^ ^)^.

One limitation is the lack of attention given to the impact of discretionary and ultra-processed foods on health and sustainability. The report states that processing foods (e.g. by partially hydrogenating oils, refining grains, and adding salt and other preservatives) can substantially affect health but this category of foods is not explicitly addressed in the healthy reference diet. There is no reason provided for excluding ultra-processed foods from the analysis, and further explanation is warranted. Further development of the healthy reference diet or its narrative is needed which specifically clarifies the inclusion of zero consumption recommendations for some nutritious foods as well as foods to avoid or limit.

It is inevitable the EAT-*Lancet* report will provoke debate given it is attempting to present such a complex topic in simple terms. However, the overall approach has been scientifically rigorous and captures the importance of needing transformative change. A key question now is, ‘Will the report lead to help to challenge and change the professional teaching and research standards, competencies, agendas and funding capacities needed to undertake the policy and practice changes required?’ This will depend on the extent to which relevant faculties will need to modify their curricula and to which funding bodies actively incentivise an integrated multidisciplinary research agenda for healthy and sustainable food systems. Positively, new research has demonstrated that nutrition science has undergone periodic historical paradigm shifts, with a new paradigm that integrates environmental sustainability now emerging^(^ ^34^ ^)^.

The scale and urgency of the health and sustainability challenges facing humanity demand food system transformation. Nudges, adjustments or tweaks on their own will be insufficient to achieve healthy diets from sustainable food systems. This is why initiatives such as the EAT-*Lancet* report are crucial to prompt the changes needed. The report presents an opportunity to launch new policy initiatives and reinvigorate policies and actions that have sought to gain traction in this area but with mixed success in the past. Yes, there are some issues to address with the report and a priority into the future will be ongoing monitoring and review of its recommendations. We encourage the public health nutrition community to embrace the approach because it will take our collaboration, commitment and contribution to achieve food system transformation by 2050.
